# Optimizing HIV prevention for women: a review of evidence from microbicide studies and considerations for gender-sensitive microbicide introduction

**DOI:** 10.7448/IAS.18.1.20536

**Published:** 2015-12-21

**Authors:** Elizabeth G Doggett, Michele Lanham, Rose Wilcher, Mitzy Gafos, Quarraisha A Karim, Lori Heise

**Affiliations:** 1 Research Utilization Department, FHI 360, Durham, NC, USA; 2 Social and Behavioral Health Sciences Department, FHI 360, Durham, NC, USA; 3 Medical Research Council Clinical Trials Unit at University College London, Institute of Clinical Trials and Methodology, London, UK; 4 CAPRISA, Nelson Mandela School of Medicine, University of KwaZulu-Natal, Durban, South Africa; 5 Department of Epidemiology, Columbia University, New York, NY, USA; 6 Department of Global Health and Development, London School of Hygiene and Tropical Medicine, London, UK

**Keywords:** microbicides, gender, gender norms, HIV prevention, woman-controlled methods

## Abstract

**Introduction:**

Microbicides were conceptualized as a product that could give women increased agency over HIV prevention. However, gender-related norms and inequalities that place women and girls at risk of acquiring HIV are also likely to affect their ability to use microbicides. Understanding how gendered norms and inequalities may pose obstacles to women's microbicide use is important to inform product design, microbicide trial implementation and eventually microbicide and other antiretroviral-based prevention programmes. We reviewed published vaginal microbicide studies to identify gender-related factors that are likely to affect microbicide acceptability, access and adherence. We make recommendations on product design, trial implementation, positioning, marketing and delivery of microbicides in a way that takes into account the gender-related norms and inequalities identified in the review.

**Methods:**

We conducted PubMed searches for microbicide studies published in journals between 2000 and 2013. Search terms included trial names (e.g. “MDP301”), microbicide product names (e.g. “BufferGel”), researchers’ names (e.g. “van der Straten”) and other relevant terms (e.g. “microbicide”). We included microbicide clinical trials; surrogate studies in which a vaginal gel, ring or diaphragm was used without an active ingredient; and hypothetical studies in which no product was used. Social and behavioural studies implemented in conjunction with clinical trials and surrogate studies were also included. Although we recognize the importance of rectal microbicides to women, we did not include studies of rectal microbicides, as most of them focused on men who have sex with men. Using a standardized review template, three reviewers read the articles and looked for gender-related findings in key domains (e.g. product acceptability, sexual pleasure, partner communication, microbicide access and adherence).

**Results and discussion:**

The gendered norms, roles and relations that will likely affect women's ability to access and use microbicides are related to two broad categories: norms regulating women's and men's sexuality and power dynamics within intimate relationships. Though norms about women's and men's sexuality vary among cultural contexts, women's sexual behaviour and pleasure are typically less socially acceptable and more restricted than men's. These norms drive the need for woman-initiated HIV prevention, but also have implications for microbicide acceptability and how they are likely to be used by women of different ages and relationship types. Women's limited power to negotiate the circumstances of their intimate relationships and sex lives will impact their ability to access and use microbicides. Men's role in women's effective microbicide use can range from opposition to non-interference to active support.

**Conclusions:**

Identifying an effective microbicide that women can use consistently is vital to the future of HIV prevention for women. Once such a microbicide is identified and licensed, positioning, marketing and delivering microbicides in a way that takes into account the gendered norms and inequalities we have identified would help maximize access and adherence. It also has the potential to improve communication about sexuality, strengthen relationships between women and men and increase women's agency over their bodies and their health.

## Introduction

HIV is the leading cause of death among women of reproductive age worldwide [1], and the incidence of HIV among women has been rising for more than a decade [2]. Women's HIV risk is driven in large part by gendered norms and structural inequalities between women and men.

In the majority of societies, men are economically and socially dominant over women, with social norms often justifying this arrangement as “natural.” Despite global patterns of inequality, gendered roles, norms and relations are manifested differently in different regions and change over time. Gendered social norms and power dynamics between women and men are influenced by global and local histories, economies and values. In many contexts, prevalent constructs of masculinity pressure men and boys to control sexual decision-making, have multiple partners and aggressively pursue sex – sometimes to the point of coercion [3]. Women are often expected to be submissive on sexual matters, hindering their ability to negotiate safer sex practices, especially in the context of marriage, violent relationships and intergenerational partnerships [3,4]. This social system also limits women and girls’ access to education, autonomous livelihoods and financial resources, which leads some to engage in transactional sex. Intimate partner violence (IPV) is pervasive in many countries [5] and is another key factor driving women's HIV vulnerability. Traditional HIV prevention approaches remain dependent on male initiation or cooperation, exacerbating women's vulnerability to HIV when they are unable to influence their partner's commitment to monogamy or to negotiate condom use.

Vaginal microbicides are products conceptualized in the early 1990s to give women increased control and agency over HIV prevention [6]. Together with oral pre-exposure prophylaxis (PrEP), these antiretroviral (ARV) drug-based products could create an HIV prevention landscape where women have choices of different formulations of HIV prevention products that they can initiate and use with or without their partners’ agreement. The CAPRISA 004 study provided proof of concept that vaginal 1% tenofovir gel reduces HIV infections by 39 percent in women and genital herpes infections by 51% [7]. However, the subsequent VOICE and FACTS 001 trials were not able to confirm the effectiveness of microbicide gels, largely due to low adherence to the gel, especially by younger women, many of whom were in unstable partnerships and/or living with their parents [8,9].

The issue of adherence is complex and likely driven by beliefs about the product's efficacy and perception of one's own risk, both of which may vary among women of different ages, relationship status and economic circumstances. For example a married woman may be more inclined to go against her husband's wishes to use a product that is 90% effective than one that is only 30% effective, and a woman may be more likely to adhere to a product if she knows her partner is HIV positive. However, in some contexts women are less likely to know their partner's HIV status than men [10]. These are just a few of the gendered norms that can affect women's motivations, willingness and ability to adhere to a gel regimen.

The future of microbicide gel is uncertain, but the results of the FACTS 001 and VOICE trials underscore the need for continued research into HIV prevention options that work for women. Similarly, FEM-PrEP and VOICE were not able to demonstrate the effectiveness of oral PrEP among women. We are still learning what works for women in different circumstances. The CAPRISA 008 study, an open-label study testing the use of 1% tenofovir gel accessed in family planning clinics may give us more insight into how well women adhere to gel when they know the product is effective. Additionally, we are awaiting results from trials testing delivery of ARV drugs through vaginal rings worn continuously and changed monthly. Regardless of the direction the ARV-based HIV prevention field takes, we can learn a great deal from examining gender issues raised in social and behavioural studies of vaginal microbicides, as these same gender dynamics will likely affect the acceptability and use of other woman-initiated HIV-prevention products. This review aims to identify gender norms that are likely to facilitate or pose barriers to women's access to and use of ARV-based prevention products outside of the trial context.

## Methods

We conducted PubMed searches for microbicide studies published in journals between 2000 and 2013. Search terms included trial names (e.g. “MDP301”), microbicide product names (e.g. “BufferGel”), researchers’ names (e.g. “van der Straten”) and other relevant terms (e.g. “microbicide”). We included microbicide clinical trials; surrogate studies in which a vaginal gel, ring or diaphragm was used without an active ingredient; and hypothetical studies in which no product was used. Social and behavioural studies implemented in conjunction with clinical trials and surrogate studies were also included. Although we recognize the importance of rectal microbicides to women, we did not include studies of rectal microbicides, as most of them focused on men who have sex with men. Using a standardized review template, three reviewers read the articles and looked for gender-related findings in key domains (e.g. product acceptability, sexual pleasure, partner communication, microbicide access and adherence).

We anticipated differences in the gender issues based on the type of study because women who participate in surrogate studies or clinical trials actually experience the challenges of using a product while women in hypothetical studies can only imagine the issues they may face. We also anticipated differences among geographical regions, study population or product formulation. However, we found the gender-related results to be surprisingly similar across study types, products, populations and locations. Any notable differences, as well as instances where data comes primarily from one study type, are detailed in the findings. Additionally, Tables 1 through 3 present the included studies by study type, product, population and location.

**Table 1:** Hypothetical studies

Reference/reference number	Hypothetical product	Countries	Study population	Method
Becker [71]	Not specified	South Africa	38 IDIs; 23 FGDs with women and men	Qual
Bisika [34]	Gel	Malawi	32 women; 55 men	Qual
Coggins [26]	Gel	Mexico, USA, Zimbabwe	90 men	Qual
Hammett [75]	Gel	USA, Puerto Rico	743 women	Quant
Hoel [25]	Not specified	South Africa	29 women	Qual
Kohli [16]	Gel	India	15 men	Qual
Lees [23]	Gel	Tanzania	Ethnographic observation of 1573 women; approximately 20 men. Six FGDs with women; two with men; 8–12 participants in each group	Qual
Montandon [56]	Gel	Kenya	30 adolescent girls plus some 31 mothers and community leaders; 28 fathers and community leaders	Qual
Ramjee [70]	Gel	South Africa	243 men	Qual
Terris-Prestholt [81]	Gel	South Africa	22 women	Mixed
Orner [17]	Gel	South Africa	213 women	Qual
Tolley [60]	Gel	India	30 women; 15 men	Qual
van der Straten [54]	Diaphragm	Zimbabwe	75 women	Quant
van de Wijgert [27]	Gel	Zimbabwe	43 men	Qual
Veldhuijzen [35]	Gel	Rwanda	Seven FGDs with approximately 80 women and men	Qual
Woodsong [72]	Topical microbicides	India, Malawi, South Africa, Tanzania, Zambia, Zimbabwe, USA	Not specified (review of three studies)	Qual

IDI, In-Depth Interview; FGD, Focus Group Discussion; Qual, Qualitative; Quant, Quantitative.

**Table 2:** Surrogate studies

Reference/reference number	Surrogate product	Countries	Study population	Method
Green [59]	Female condom, foaming tablets, contraceptive sponge, Delfen foam, film and gel	Uganda	131 women; 21 men	Qual
Jones [33]	Astroglide Silken Secret (high-viscosity gel), KY Jelly (low-viscosity gel), Lubrin (suppository)	Zambia	301 women	Quant
Martin [15]	Gel	Thailand	23 women; 28 men	Qual
Montgomery [20]	Gel	South Africa, Tanzania, Uganda, Zambia	45 couples	Qual
Pool [48]	Female condom, foaming tablets, contraceptive sponge, Delfen foam, film and gel	Uganda	138 women; 42 men	Qual
Salter [43]	Gel	Malawi	1686 women; 21 men	Mixed
Tanner [21]	Silken Secret vaginal moisturizer	USA	40 women	Qual
van der Straten [61]	Ring	South Africa, Tanzania	157 women; 19 men	Qual
Weeks [74]	Vaginal moisturizer	USA	546 women interviewed/surveyed; 94 participating in surrogate trial	Mixed

Qual, Qualitative; Quant, Quantitative.

**Table 3:** Microbicide clinical trials

Reference/reference number	Product	Countries	Study participants	Method
Abaasa [58]	Gel	Uganda	544 women	Quant
Abdool Karim [7]	Gel	South Africa	889 women	Quant
Behets [57]	Gel; diaphragm with gel	Madagascar	314 women	Quant
Bentley [28]	Gel	Zimbabwe, Malawi, India, Thailand	99 women and men	Mixed
Carballo-Dieguez [36]	Gel	USA	21 men	Mixed
Carballo-Dieguez [24]	Gel	USA, Puerto Rico	69 women	Mixed
Gafos [12]	Gel	South Africa	136 women; 61 men	Qual
Gafos [37]	Gel	South Africa	34 women	Qual
Gafos [69]	Gel	South Africa	1092 women	Mixed
El Sadr [46]	Gel	USA	59 women; 11 men	Quant
Greene [38]	Gel	Uganda, Benin and India	53 women	Qual
Guest [18]	Diaphragm with gel	South Africa	120 women	Mixed
Hoffman [41]	Gel	USA	79 women	Qual
Kacanek [19]	Diaphragm with gel	South Africa, Zimbabwe	206 women; 41 men	Qual
Kacanek [76]	Diaphragm with gel	South Africa, Zimbabwe	4505 women	Quant
Lanham [62]	Gel, ring	South Africa, Kenya, Tanzania	535 interviews, 107 focus groups with men and women	Qual
Marrazzo [8]	Gel and oral PrEP	South Africa, Uganda, Zimbabwe	5029 women	Quant
Mantell [39]	Gel	South Africa	94 women	Qual
Mngadi [65]	Gel	South Africa	846 women	Quant
Montgomery [14]	Gel	South Africa, Zambia, Tanzania and Uganda	464 women	Qual
Montgomery [64]	Gel	Zimbabwe, South Africa	2452 women	Quant
Montgomery [31]	Diaphragm with gel	Zimbabwe	103 women	Quant
Montgomery [32]	Diaphragm with gel	Zimbabwe	955 women	Quant
Muchomba [67]	Oral PrEP, gel	Multicountry; not specified	47,157 women and men	Quant
Mzimela [50]	Gel	South Africa	33 women and men	Qual
Pistorius [40]	Gel	South Africa	64 women	Qual
Ramjee [22]	Gel	South Africa	40 women; 37 men	Mixed
Ramjee [44]	Gel	South Africa	40 women; 20 men	Mixed
Rosen [41]	Gel	USA	79 women	Mixed
Sahin-Hodoglugil [66]	Diaphragm with gel	South Africa and Zimbabwe	2316 women surveyed, 104 in FGDs; 37 men	Mixed
Sahin-Hodoglugil [30]	Diaphragm with gel	Zimbabwe and South Africa	105 women; 41 men	Qual
Stadler [45]	Gel	South Africa	179 women (+42 FGDs with women trial participants and community members); 42 men (+42 FGDs with women trial participants and community members)	Qual
Stadler [49]	Gel	South Africa	150 women	Mixed
Vandebosch [73]	Gel	Côte d'Ivoire; Benin; South Africa; Thailand	764 women	Quant
van der Straten [63]	Diaphragm with gel	Zimbabwe	117 women	Quant
Venables [42]	Gel	South Africa	175 women; 82 men	Mixed
Whitehead [68]	Gel	Thailand	271 women	Quant
Woodsong [13]	Gel	Malawi	321 women (+81 community stakeholders and health providers); 109 men (+81 community stakeholders and health providers)	Qual

PrEP, pre-exposure prophylaxis; FGD, Focus Group Discussion; Qual, Qualitative; Quant, Quantitative.

After presenting the results from the literature review, we discuss the implications for future microbicide research and potential microbicide introduction, including marketing, service delivery, counselling and support for women and community education. We also explore potential effects of microbicide introduction on women's empowerment and gender relations.

## Results and discussion

The gender norms, roles and relations that will likely affect women's ability to access and use microbicides fall into two broad categories: 1) norms related to women's and men's sexuality and 2) power dynamics within intimate relationships. We also discuss the ways in which microbicide introduction may affect the relative status of women and men.

### Norms related to women's and men's sexuality

Though norms about women's and men's sexuality vary among cultural contexts, women's sexual behaviour and pleasure are typically less socially acceptable and more restricted than men's [11–14]. For example, in many contexts, including parts of sub-Saharan Africa and Southeast Asia where the majority of the microbicides studies have been conducted, women are expected to be virgins before marriage and monogamous within marriage. In contrast, it is often accepted as inevitable and even considered masculine for men to have multiple partners regardless of marital status [15–19]. Similarly, women – especially young women – are expected to be naive and passive in relation to sexuality [11], which impacts women's negotiation of the frequency and safety of sex, as well as their experience of sexual pleasure. Thus, sexual norms related to women's age, relationship or marital status, sexual pleasure and other sexual preferences and practices, such as so-called dry sex, affect the acceptability and use of microbicides, including perceptions of who is an appropriate microbicide user.

#### Sexual norms, risk perception and microbicide user groups

In microbicide trials and hypothetical studies, women's, men's and couples’ opinions were mixed about which women are most at risk, which influences both perceptions of the appropriate user group for microbicides as well as adherence to microbicides. In several studies, women in steady partnerships, including married women, were perceived to benefit most from microbicides [15–22], because so many married women have limited control over negotiating sex and condom use [15–19,23]. As discussed below, this difficulty stems in part from issues of trust and intimacy within primary or married relationships. In other studies, married women were not perceived to be at risk for HIV and therefore microbicides were viewed as unnecessary within marriage [15,24,25]. In these studies, respondents felt that sex workers, women with casual sex partners or women in HIV serodiscordant relationships were the most appropriate user groups for microbicides [15–17,26–28]. However, respondents in one microbicide surrogate study predicted that if microbicides are promoted only to specific high-risk groups such as female sex workers, the product could be perceived to be linked to infidelity and risky behaviours [20], potentially stigmatizing microbicides and precluding married women and adolescents from using them.

In addition to product acceptability, we know from the oral PrEP literature that risk perception is positively associated with product adherence [29]. Unfortunately, due to the gendered sexual expectations discussed above, many women's primary risk is via their main or stable partner. Because women may be less likely than men to know their partner's status, it can be difficult for women to estimate their risk accurately [10].

#### Sexual pleasure

The potential for promoting sexual pleasure – for women and men alike – is a distinct advantage of vaginal microbicide products. Sexual pleasure is highly gendered, such that men's pleasure often takes precedence over women's pleasure [13,21,30–32]. This disparity has implications for how microbicides are marketed and for whether people will choose to replace condoms with microbicides or use the two methods together.

Increased sexual pleasure from the additional lubrication in gel formulations and increased libido from product use positively influenced the acceptability of microbicides [13,33–35]. Most microbicide trials found that the gel increased sexual pleasure [15,16,18,24,28,30,36–45] or at least did not change sexual pleasure [24,28,41,46,47]. This finding was true across study populations – including sex workers, women and men with steady partners and HIV-positive and HIV-negative people. It should be noted that, though acceptability was sometimes influenced by both female and male sexual pleasure [12,14,37,48], a stronger predictor of acceptability in some studies was a male partner's sexual pleasure, and women's experiences of sexual pleasure often referred to lack of pain during coitus or to pleasuring their partner [13,21,30–32]. In a study from South Africa, some women reported that they touted the potential for increased sexual pleasure in order to convince their partners to agree to their use of the product and to reduce the likelihood of their partners’ negative or violent reactions [49].

Many HIV prevention experts have expressed concern that the introduction of microbicides might further discourage people from using condoms, which offer greater HIV protection on a per-sex-act basis, added protection from other STIs and pregnancy and are likely less expensive than microbicides. Indeed, some women and men in trials, surrogate studies and hypothetical studies felt that a microbicide gel was preferable to condoms because of the greater sexual pleasure it offers [15,16,41,44,45]. In other studies in which both gel and condoms were used, some female and male participants indicated that the increased sexual pleasure offered by the gel balanced the decreased sexual pleasure experienced with condom use and hence enabled both condoms and gel to be used simultaneously [18,37,41,50]. Furthermore, consistent microbicide use may provide a woman more HIV protection over time than inconsistent condom use. Evidence suggests that the decision to use condoms is dominated by men, and it is not clear what impact the availability of an effective microbicide will have on the power dynamics and decision-making related to condom use [51,52]. Public health experts have discussed the possibility of promoting microbicides for use in relationships or individual sex acts where women are unable to negotiate condom use.

#### Sexual preferences and related intravaginal practices

The perception that men often prefer what is termed “dry” or “tight” sex also has implications for microbicide acceptability. However, the term *dry* is likely a misnomer: a multicountry study found that practices to enhance sexual pleasure “are not always aimed at ‘drying’ the vagina, but rather at ‘closing, warming and tightening’ the vagina” [53]. In some studies where dry sex was the stated preference, women feared that men would not like the added lubrication from a microbicide gel [13,34]. Because vaginal wetness is often linked to accusations of sexual promiscuity, infidelity or masturbation, there is a risk that lubrication from the microbicide gel could lead to accusations of impropriety [16,20,34,36] and even end a relationship [26].

However, for the most part, these fears were not realized among microbicide users. Two clinical trials in South Africa and Malawi found that women and men enjoyed the gel's lubrication, even among a minority of women who were initially concerned about the gel increasing vaginal wetness [12,13]. Other trials and surrogate studies in Malawi, South Africa, Zambia, Tanzania and Uganda found that the use of vaginal gels resulted in gains in sexual pleasure that were previously sought from other types of intravaginal insertions [12,14,43,45,54]. One surrogate gel study in Tanzania was an exception in that some men disliked the added lubrication and this influenced product use [20].

Women who have experience with vaginal insertion, usually in the form of inserting herbs and other products into the vagina to promote pleasure – in particular men's pleasure – during sex [55] could find microbicide use easier and more acceptable, in comparison with women who are inexperienced with vaginal insertion [56].

### Women's power within intimate relationships

Women's power to negotiate the circumstances of sex will impact their ability to access and use microbicides. This includes practical matters like women's lack of control over the timing of sex or the privacy to store and insert the gel, the dilemma of whether to discuss use of microbicides with male partners and the risk of experiencing IPV when using microbicides. Moreover, men's role in women's effective microbicide use can range from opposition to non-interference to active support.

#### Power to control timing of sex and privacy

In many areas, men are the primary decision-makers about sexuality; thus, women cannot always control or predict the circumstances of their sex lives. Women participating in microbicide trials, their male partners and people interviewed about potential microbicide use in Benin, India, Malawi, South Africa, Thailand, Uganda, the United States and Zimbabwe noted that women's lack of privacy to insert the gel [16,28,38,44,57,58] and limited ability to control the timing of sex (and thus the ability to apply the gel before sex) [13,34,38,41,44,59,60] may interfere with women's adherence to a coitally dependent microbicide regimen. A hypothetical study among adolescent girls, their parents and other community leaders in Kenya found the timing of gel insertion would likely be a challenge among adolescent girls, whose sex lives were described as unpredictable, rushed and illicit [56]. A ring formulation may pose fewer challenges related to privacy and timing of sex, since the ring can remain in the vagina for a month after it is inserted. Indeed, participants in a placebo ring study in South Africa and Tanzania reported discreetness, convenience and being able to leave the ring in for four weeks as favourable attributes of this product [61].

#### Male partner engagement and communication

Male partners and partnership dynamics will likely play a major role in microbicide acceptability and women's ability to use a gel product regimen [62]. In trials and surrogate studies, women's willingness to use microbicides and ability to adhere to product regimens were often influenced by women's perceptions of their partner's acceptance of the products [31,32,43,45,63–66]. It should be noted that men's involvement in microbicide use can range from constructive to coercive. Female and male respondents in trials, surrogate studies and hypothetical studies found that some men supported adherence in a constructive way, for example by reminding their partners to use the gel or helping them to insert it [14,20,32,38,40,62,64], whereas others were more coercive, demanding that their partners use the gel [20,40,62].

Women's ability to use microbicides will likely vary in different kinds of relationships. A literature review of 14 microbicide trials found that one of the most frequently cited reasons for non-adherence was having sex with steady partners as opposed to casual or paying partners [67]. Likewise, trial participants in Benin, Uganda and India – many of whom were involved in sex work – reported that adherence to the gel regimen was easier with casual partners than with steady partners [38]. These differences are likely linked with trust issues within intimate relationships, as well as women's sexual negotiating power, a factor found to be associated with consistent gel use in a microbicide gel safety trial in India [60].

A key characteristic of microbicides is that women may be able to use them without their partners’ knowledge. Studies have confirmed that women value having a product they can use without communicating with their partner [19,38,41,46–48,61,64,68]. However, study findings also suggest that many women will likely talk with their partners about using microbicides. In all clinical trials and surrogate studies that asked about partner communication, participants typically talked with steady partners about their microbicide use at some point during study participation [12,14,30,32,37,42,61,66,69].

Relationship type also affects whether and how women communicate with their partners about microbicide use. Women and men in steady relationships preferred joint decision-making on microbicide use [15,20,25,26,28,36,44,47,60,66,70–72]. In steady relationships, using microbicides without partner communication may imply infidelity or mistrust [15,35,61,66] or be perceived as challenging male authority and decision-making [16,72]. Use of a microbicide gel without partner communication may be more acceptable and feasible in casual or new partnerships, in which perceived HIV risk is likely to be highest [13,35,36,41,73]. Using a microbicide without a partner's knowledge was more common among trial participants in casual relationships and sex workers [38,66]. Women noted that a change in the amount of lubrication from the gel may be more noticeable to a steady partner than to a casual partner [12,15,20,36,38,47]. Nonetheless, many women and men in trials and hypothetical studies in sub-Saharan Africa, and in particular South Africa, acknowledged the reality that some women in steady partnerships, including married women, were likely to use the product without telling their partners [13,17,25,44].

A woman's decision to communicate with her partner about microbicides may differ by product formulation. Most of the evidence regarding partner communication about microbicides comes from gel trials; a woman may feel less of a need to discuss use of a microbicide ring or injectable with her partner because these products may be less noticeable to the partner. More evidence is needed about whether and how women communicate with their partners about microbicide rings and injectables. Finally, though the literature has not discussed women's negotiating power in relation to levels of product efficacy, the efficacy of a product is also likely to influence whether and how a woman discusses the product with her partner as well as her negotiating power if she does decide to discuss it.

#### Intimate partner violence

The perpetration of violence against women by intimate partners is pervasive around the world and is a major contributor to ill health – including HIV – among women [5]. Although particular attention needs to be paid to the role of violence in women's lives, the literature has not explored the effect of violence on women's microbicide use in much depth. Some women may fear or experience violent reactions from their partners if they bring up the subject of microbicides or use microbicides without their partners’ knowledge. IPV may also affect women's microbicide adherence and the likelihood of substituting microbicides for condoms, though the specific dynamics of these relationships are not well understood.

About 40 percent of women participating in in-depth interviews at one gel trial centre in South Africa reported experiencing IPV during the trial. More than half of the violent episodes were related to the woman's trial participation, specifically partners’ dissatisfaction with the gel and disapproval of trial participation and trial procedures, including required condom use [49]. In surrogate studies, women's willingness to use microbicides was negatively influenced by a history of male violence [74,75].

Deciding whether to talk to a partner about microbicide use may be more complicated for women in violent or abusive relationships. If a woman tries to use a gel product without her partner's knowledge and her partner finds out, he may react violently [13,30,61,69,71]. Some women may decide to communicate with their partner about microbicide use because they fear a negative or violent reaction if their partner discovers they have been using a product without discussing it first [20,69].

It also seems that some women in abusive relationships find condom use and microbicide adherence more challenging. Sex workers in Thailand reported that gel adherence was more difficult with violent clients [38]. Similarly, experience of partner violence among women in a diaphragm trial in South Africa and Zimbabwe was closely associated with non-adherence to both condoms and the diaphragm [76]. Finally, a trial of diaphragms with microbicides in Zimbabwe found that women who experienced domestic violence were more likely to substitute diaphragms with microbicides for condoms than women who had not [63].

### Microbicides and women's status

Though microbicides themselves will not empower women, microbicide introduction has the potential to increase women's control over their health and sexuality and to be a vehicle for promoting couples’ communication and improving their relationships. For example, several gel trials and one surrogate gel study found that partner communication about microbicide use can have a range of benefits including increased communication about sex, increased pleasure and intimacy, improved relationship dynamics, shared responsibility for protection and increased self-reported microbicide adherence [20,38,40,42]. Even though gendered relationship dynamics often put women at a disadvantage overall in negotiating HIV protection, in-depth interviews with women revealed that many are highly resourceful in managing relationship dynamics and often find creative ways to justify microbicide use [69]. Likewise, some women participating in surrogate studies expressed that having ownership of the product and information about it gave them some degree of power in negotiating microbicide gel use [20,21,59]. A trial of a diaphragm with gel in South Africa and Zimbabwe and a gel trial in South Africa found that although many trial participants decided to discuss microbicides with their partner initially, in many circumstances ongoing use was the woman's decision [66].

### Implications for future research and product introduction

Understanding how gendered roles and norms play into women's ability to use vaginal microbicides is critical to advancing research and informing the introduction of a range of woman-initiated HIV prevention products. We know that gender inequality impacts all women, but not every woman experiences gender inequality in the same ways, especially in different life stages, relationships and other circumstances. Ultimately, women need an array of products to choose from, guidance on matching their needs with a product and support for optimizing the protection a product offers. The findings from our review offer insights for product design, specifically which types of products may work best for women in different circumstances. They also should inform future trials of ARV-based prevention methods, including how to provide adherence counselling for women and how to engage male partners. As woman-initiated HIV prevention products make their way to markets, these findings can help policy makers and programme designers position and deliver the products in ways that maximize access, adherence and more realistic risk perception. They also suggest ways to leverage product introduction to promote open communication about sexuality, improve relationships between women and men and increase women's agency over their bodies and health.

Given the low adherence to study product in the VOICE and FACTS 001 trials, we need to better develop and match women with products that they can use successfully. For example, young women might have difficulties with a coitally dependent method like a vaginal gel because their sexual relationships may be unpredictable or because they lack the privacy to insert a product before sex; they may be more able to adhere to a longer-acting product like a ring or injectable product. On the other hand, a coitally dependent gel might be ideal for older or married women who are better able to predict when they will have sexual intercourse, who may be more likely to discuss and make decisions about microbicide use together with their partners or who may appreciate the lubricating qualities of the gel.

Microbicide acceptability could be promoted in clinical trials and potential product rollout by 1) acknowledging that many women – including married women and adolescent women – are at risk of HIV, but also being culturally sensitive to fears about infidelity and trust; and 2) engaging communities – including men – to raise awareness about and broad acceptance of the product and its value to women and families in the community.

Social norms around female sexuality that encourage women to remain naive about sex and submit to male authority in sexual encounters are even more pronounced among young women. Many young women have limited power to enforce sexual consent and to negotiate safety and pleasure. The same issues hinder parents’ and healthcare providers’ acceptance of adolescent girls’ sexuality and need for information and HIV protection. Moreover, developmentally adolescents may have difficulties accurately assessing their risk [77], planning ahead or controlling impulses. Results from FACTS 001 and VOICE showed that young women had more challenges with adherence than older women, especially when they did not have a single/primary partner and when they lived with their parents [78]. In these situations, women often lack privacy and the ability to anticipate when they will have sex. Although adolescent girls are a key population at high risk of acquiring HIV infection in many settings, few microbicide trials have included adolescents. The FACTS 002 trial, which will test the safety and acceptability of tenofovir gel among adolescent women in South Africa, will contribute more evidence in this area [79]. Additional research with adolescent girls – including enrolling them in trials and demonstration projects – is needed to ensure ARV-based HIV prevention products are acceptable and easy to use among adolescents.

As with other health services and commodities – like condoms and contraception – gender inequality is also likely to limit women's access to an ARV-based prevention product. The CAPRISA 008 study will simulate more “real world” delivery of microbicides and help identify barriers women may face to accessing the product in public-sector facilities [80]. Integrating microbicides or other woman-initiated prevention products into services women already attend, such as family planning and prenatal care, may increase access. Adolescents may prefer to access products through other youth-friendly services, if they are available. Given some women's limited access to resources, ARV-based prevention products will also need to be affordable – either heavily subsidized or free – so that cost does not create a major barrier to access.

A microbicide programme will also need to recognize and address complex gendered norms and practices in relation to sexual pleasure and traditional intravaginal practices. For example, including marketing messages about microbicides’ potential for increased sexual pleasure as part of a broader marketing strategy may make the product appealing to many. However, this strategy is unlikely to be optimal in all settings given that women's sexual pleasure is still taboo in some contexts [81]. Programme designers should use such marketing strategies only when culturally appropriate and pretest all marketing messages. Finally, marketing messages about sexual pleasure should strive to be sex-positive and carefully challenge social norms that prioritize male sexual pleasure and condemn female sexual pleasure.

Although it may not be realistic to expect widespread dual use of microbicides and condoms, we need to ensure that women who already are successfully negotiating condom use do not lose their ability to do so with the availability of other HIV prevention products. Gendered norms that affect how feasible it is for a woman to insist on condom use may vary over the course of a woman's life and in different relationships, and in fact women's experiences in microbicide trials have highlighted the creativity that women can bring to managing everyday relationship dynamics. Woman-initiated prevention products could be promoted for use by women in contexts where they are not able to negotiate condom use, such as within marriage and other long-term partnerships, while continuing to promote condoms in casual relationships and for transactional or paid sex. On an individual level, providers can help women and couples determine the most appropriate HIV prevention method for their situations, keeping in mind that microbicides are likely to be less effective than condoms but, if used consistently, are likely to provide more HIV protection than inconsistent condom use [82].

A woman's decision about whether and how to communicate about microbicides is complex. We need to determine how to support a woman in this decision in a way that promotes greater self-determination, improves couples’ communication and anticipates any negative consequences a woman might experience from her partner. Counselling can help women strategize about how to talk with their partners if they want to and what to do if a woman's partner discovers she has been using microbicides or another prevention method without his knowledge. Further, microbicide trials and eventually microbicide programmes should consider how to constructively engage men so they can support – or at least not actively impede – their partners’ product use when such support is desired by the woman. Promoting men's awareness of woman-initiated HIV-prevention products through community education could make it easier for couples to discuss the methods. However, recognizing that women have varying levels of power in relationships, strategies to engage men in product introduction must also be careful not to undermine a woman's autonomy to decide whether to use the product and whether to communicate with her partner about it [62]. Some trials are already counselling women about partner communication and working to engage men, but evaluation is needed to determine which approaches promote microbicide adherence, improve relationship quality and produce gains toward gender equality.

Few sexual health services adequately assess women's risk of IPV, but the introduction of microbicides could offer the potential to better coordinate efforts to address IPV while offering women new tools to mitigate the risk of HIV. As such, when basic services for survivors of IPV are available, healthcare providers should proactively screen for IPV, counsel women and make referrals to support services. Providers will also need to be prepared to address the potential effect of IPV on adherence and a woman's decision about whether to talk with her partner about product use.

Knowledge gaps remain about how woman-initiated HIV prevention products will affect women's status, empowerment and relationship dynamics. Given the influence of gendered norms on microbicide acceptability and adherence, future trials and programmes should measure the impact of product introduction on gender-related outcomes. Measurable outcomes could include women's increased knowledge of sexuality and HIV protection, increased couples’ communication and more gender-equitable attitudes among microbicide users and their partners. Programmes may also want to monitor whether microbicide use or negotiation triggers violent episodes, especially among women who live in situations of ongoing abuse – and if so, whether the health system effectively supports these women. Further research is necessary to understand whether, in the context of an effective product, couples will make decisions together about HIV prevention or if the burden of responsibility for HIV prevention will shift to women. Moreover, in designing programmes to mitigate the gender inequalities highlighted in this paper, it is important not to reinforce notions of women's unequal status and disempowerment.

## Conclusions

Microbicides and other ARV-based products are not a magic bullet for HIV prevention or women's empowerment. Whereas identifying effective woman-initiated products will offer an important way for women to protect themselves from HIV, such products will fall short of their potential if gender norms are not taken into account when testing and introducing new technologies. This review illustrates that gender norms will likely affect many aspects of use of woman-initiated HIV prevention products, including the degree to which they are seen as necessary and acceptable for women to use; whether women who want microbicides or other products can easily access them; whether and how women will communicate with their partners about product use and how men will respond; and how well women will adhere to the dosing regimen. However, if product introduction programmes include strategies to overcome the gender-based obstacles women may face, the programmes will have the potential to increase couples’ communication, improve relationship quality, reduce women's HIV risk, give women increased knowledge about sexuality and enhance women's power to prevent HIV.
